# A Bifunctional Phosphoglucomutase/Phosphomannomutase from *Thermococcus kodakarensis*: Biophysical Analysis and Cryo-EM Structure

**DOI:** 10.3390/biom15030319

**Published:** 2025-02-21

**Authors:** Zahra Naz, Ishan Rathore, Muhammad Saleem, Moazur Rahman, Alexander Wlodawer, Naeem Rashid

**Affiliations:** 1 School of Biological Sciences, University of the Punjab, Lahore 54590, Pakistan; zahranaz55@ymail.com (Z.N.); msaleem.sbs@pu.edu.pk (M.S.); moaz.sbs@pu.edu.pk (M.R.); 2 Center for Structural Biology, National Cancer Institute, National Institutes of Health, Frederick, MD 21702, USA; ishan.rathore@nih.gov

**Keywords:** *Thermococcus kodakarensis*, phosphoglucomutase/phosphomannomutase, three-dimensional structure, cryo-electron microscopy (cryo-EM)

## Abstract

Phosphoglucomutase (EC 5.4.2.2., PGM), a key enzyme of glycogenolysis and glycogenesis, catalyzes the interconversion of glucose 1-phosphate and glucose 6-phosphate, whereas phosphomannomutase (EC 5.4.2.8., PMM) transfers the phosphate group from the 1′ to the 6′, or from the 6′ to the 1′ position in mannose phosphate. However, in the hyperthermophilic archaeon *Thermococcus kodakarensis*, a single gene, *Tk1108*, encodes a protein with both PGM and PMM activities. Here, we report biophysical analysis and the 2.45 Å resolution cryo-EM structure of this novel enzyme. Our results demonstrate a specific arrangement of the four subunits in the quaternary structure, displaying a distinct catalytic cleft required for the bifunctional activity at extremely high temperatures. To the best of our knowledge, this is the first biophysical characterization and cryo-EM structure elucidation of a thermostable, bifunctional PGM/PMM.

## 1. Introduction

Glycogenolysis and glycogenesis are essential processes in maintaining energy homeostasis. Glycogenolysis is the process of breaking down glycogen_(n)_ to glucose 1-phosphate and glycogen_(n−1)_, whereas glycogenesis is the process of glycogen synthesis for storage purposes [1]. The main enzyme active in these two pathways is phosphoglucomutase (PGM) (EC 5.4.2.2.), which metabolically catalyzes the interconversion of glucose 1-phosphate (G1P) to glucose 6-phosphate (G6P) [2]. G1P, the product of intracellular polysaccharide degradation catalyzed by glycan phosphorylases, is a key metabolite for glycolysis regulation [3] to generate ATP, NADH, and biosynthetic precursors (pyruvate or 3-phosphoglycerate). Alternatively, G1P is used as a precursor of sugar-nucleotides that are used for building polysaccharides essential for energy storage in the cell. These reactions connect the central carbon metabolism with the synthesis and degradation of glycogen [2,4]. A divalent metal ion, usually Mg^2+^ or Mn^2+^, acts as activator for enzymatic activity [3].

The hyperthermophilic archaeon *Thermococcus kodakarensis* displays heterotrophic growth on a variety of organic substrates, such as amino acids, pyruvate, and starch [5]. Although phosphoglucomutase activity has been detected in the crude extract of *T. kodakarensis* cells, no open reading frame was annotated as PGM in the genome databases [6]. However, the genome sequence contains four open reading frames (TK1108, TK1404, TK1777, and TK2185) annotated as phosphomannomutase (PMM) genes [7]. All four open reading frames are members of cluster 1109 of orthologous genes (COG1109), and it has been found previously that *TK1777* encodes a phosphopentomutase [8]. Among the remaining three gene products, only that of *Tk1108* exhibited both phosphoglucomutase and phosphomannomutase activities; based on its former function, the protein was named PGM*_Tk_*. Phosphoglucomutase activity of PGM*_Tk_* has been evaluated with substrates such as glucose 1-phosphate (G1P), mannose 1-phosphate (M1P), and 1-deoxyribose 1-phosphate (dR1P). A high level of activity was seen with G1P (690 μmol min^−1^ mg^−1^). Moreover, thermostability studies confirmed a half-life of 85 min at 100 °C [6].

Until now, crystal structures of three archaeal, eight bacterial, and seven eukaryotic PGMs have been determined (https://www.rcsb.org/; accessed on 11 July 2024). Archaeal enzymes include the PGM from *Pyrococcus* sp. strain ST04 (4UW9) [9], *Sulfolobus tokodaii* (2F7L) [10], and *Pyrococcus horikoshii* (1WQA; PGM*_Ph_*) [11]. However, there is no report on a three-dimensional structure of phosphoglucomutase from the archaeal genus Thermococcus. Although three-dimensional structures can now be predicted using advanced computational algorithms such as AlphaFold2 [12], experimental structure determination is still necessary in order to broaden the available experimental database that informs these structure prediction tools. Considering the high homology towards phosphomannomutases and high mutase activity towards phosphoglucose, the objective of the present study was to experimentally determine the three-dimensional structure of PGM*_Tk_* that would enable a better understanding of its biochemical and enzymatic properties. The present study describes the biophysical analysis, as well as elucidation of the three-dimensional structure of this novel enzyme using cryo-EM.

## 2. Materials and Methods

### 2.1. Construction of the Expression Vector

The PGM construct pET-His_6_-TEV-Tk1108 was obtained by introducing nucleotides for His_6_-TEV (tobacco etch virus protease cleavage site) upstream of the PGM gene in the pET-21a(+)-Tk1108 construct using the Quick-change reaction (Figure S1). The forward (5′-CTTTAAGAAGGAGATATACATATGGGCAGCCATCATCACCATCATCACGAGAACCTG-3′) and reverse (5′-CGCCAAACGTTCCAAAGAGCTTTCCCTGGAAGTACAGGT TCTCGTGATGATGGTGATG-3′) primers for the PCR were obtained from Integrated DNA Technologies, USA. The product of the first PCR was purified (QIAquick PCR Purification Kit) to be used as the primer for the second reaction. Following another round of purification, *Dpn*I digestion was performed to remove methylated DNA, yielding the newly synthesized construct. The recombinant construct was used to transform *E. coli* DH5α cells (Agilent Technologies, Santa Clara, CA, USA), followed by confirmation of clone by restriction digestion (Figure S2), and sequencing (Figure S3).

### 2.2. Heterologous Production and Partial Purification of Recombinant PGM_Tk_

*E. coli* Rosetta^TM^ 2(DE3)pLysS cells were transformed with a pET-21a(+)-His_6_-TEV-Tk1108 plasmid. A single colony from the transformed cells was cultured overnight in LB broth with ampicillin (final concentration 50 µg/mL) and chloramphenicol (final concentration 30 µg/mL) at 37 °C, 200 rpm. The following day, this culture was diluted into fresh LB broth and incubated until the OD_600_ reached 0.6. Gene expression was induced with 1 mM isopropyl ß-D-1-thiogalactopyranoside (IPTG, Fisher Scientific, Waltham, MA, USA) followed by a 4 h post-induction incubation at 37 °C, 200 rpm. Afterward, cells were collected by centrifugation, washed, and resuspended in lysis buffer (50 mM Tris-HCl, pH 8.0 + 500 mM NaCl). Cell lysis was performed via sonication (10 s on, 30 s off, 45% amplitude) in an ice bath, and the lysate was centrifuged at 60,000× *g* for 20 min at 10 °C to separate the soluble and insoluble fractions. The soluble fraction was subjected to heat lysis at 85 °C for 25 min, followed by ice incubation for 45 min, which allowed for the removal of heat-labile proteins of *E. coli* by centrifugation at 60,000× *g* for 30 min at 10 °C. Both soluble and insoluble fractions were analyzed on SDS-PAGE (Figure S4).

The soluble fraction was filtered with a 0.45 µm membrane and loaded on a Ni-NTA affinity chromatography column (HisTrap^TM^ HP, Cytiva^TM^, Marlborough, MA, USA) pre-equilibrated with Buffer A: 50 mM Tris-HCl, pH 8.0 + 500 mM NaCl (Bio-Rad NGC chromatography system). The unbound and non-specific proteins were removed by a wash with 20 column volumes (CVs) of Buffer A. The bound His-tagged PGM*_Tk_* was eluted with an isocratic gradient (50%) of Buffer B (500 mM imidazole + 50 mM Tris-HCl, pH 8.0 + 500 mM NaCl). Eluted fractions containing significant recombinant protein were pooled and concentrated by using an Amicon^®^ Ultra-15 Centrifugal Filter Units (Merck Millipore, Burlington, MA, USA). The concentrated sample was loaded onto a HiLoad^®^ 16/60 Superdex*^®^* 200 pg gel filtration column (Cytiva^TM^, Marlborough, MA, USA) equilibrated with 50 mM HEPES pH 7.0 + 200 mM NaCl. The eluted sample was analyzed by denaturing and non-denaturing PAGE to assess the purity of the protein.

### 2.3. Mass Spectrometry Studies

Molecular integrity and sample composition were evaluated using mass spectrometry in positive electrospray ionization (ESI-MS) mode using the Agilent 6130 Single Quadrupole LC/MS System. The sample was prepared by diluting protein (3 mg/mL) in 20% *v*/*v* isopropanol: 10% *v*/*v* acetic acid and 70% *v*/*v* water. The prepared sample was loaded on a Poroshell StableBond 300 C3 column via a syringe pump. The analysis was performed in mobile phase comprising solution A (5% *v*/*v* acetic acid) and solution B (95% *v*/*v* acetonitrile) at 40 °C, with a capillary voltage of 3.5 kV. High-purity nitrogen was used as an auxiliary and nebulizing gas. The obtained data were subjected to deconvolution using optimized maximum entropy algorithms.

### 2.4. Secondary Structure Analysis Using CD Spectrometry

The protein sample was diluted to 0.3 mg/mL in 5 mM Tris-HCl, pH 8.0, and CD spectrum was recorded in a temperature range of 40–90 °C using a Jasco Circular Dichroism Spectrophotometer (Easton, MD, USA) over a wavelength range of 200–260 nm in a 1 mm cuvette. The final spectrum was obtained after subtracting the buffer spectrum from the sample one. The mean residual weight (MRW) of the protein was used to estimate the mean residual ellipticity (MRE). The obtained scan was analyzed using an online program, DichroWeb (http://dichroweb.cryst.bbk.ac.uk/html/home.shtml; accessed on 11 August 2024) [13].

### 2.5. Mass Photometry Studies

The oligomeric nature of PGM*_Tk_* in solution was assessed by mass photometry. The protein was diluted to three different concentrations, 23 nM, 65 nM, and 133 nM, in a freshly prepared and degassed buffer (50 mM HEPES, pH 7.0 + 200 mM NaCl). Mass photometry [14] data were acquired on a Refeyn Two MP using Acquire MP ver. 2024 R1 (Refeyn Ltd., Oxford, UK) software. The results were generated as video data and analyzed by the DiscoverMP program (Refeyn Ltd., Oxford, UK). A standard graph was obtained from measurements of thyroglobulin (670 kDa) and β-amylase (56, 112, 224 kDa). A standard mass calibration with a bin width of 5 kDa was utilized to convert the raw contrast values to molecular mass.

### 2.6. Dynamic Light Scattering (DLS) Analysis

The hydrodynamic radius and the overall homogeneity of PGM*_Tk_* particles were evaluated by DLS analysis [15]. The protein sample was diluted up to a 100 µM final concentration in 50 mM HEPES, pH 7.0 + 200 mM NaCl. The buffer alone was used as a blank and was loaded along with the sample on the standard plate (Greiner Bio-one 1536 well plate; Greiner, Monroe, NC, USA). To avoid any false results, air bubbles were removed by gentle tapping and centrifugation. A scan was performed on a DynaPro Plate Reader (Wyatt Technology Corporation, Goleta, CA, USA) at 22 °C with an acquisition time of five seconds and default parameters, collecting 20 data points per scan. The obtained data were analyzed using the DYNAMICS program (Wyatt Technology Corporation, CA, USA).

### 2.7. Differential Scanning Fluorimetry

Differential scanning fluorimetry was carried out in order to analyze the stability of heterologously produced PGM*_Tk_* at pH 7.0, 8.0, and 9.0. The melting temperature of the protein was measured as the ratio of tryptophan fluorescence at 330 and 350 nm, after UV excitation at 280 nm [16]. The protein sample was diluted up to 2 mg/mL in buffer (50 mM HEPES pH 7.0 + 200 mM NaCl) and placed in glass capillaries with 10 µL of each sample. Scans of fluorescence from 20 °C to 95 °C at a rate of 1 °C/min were performed with Prometheus NT.48 (NanoTemper Technologies, Munich, Germany). Data were obtained from an average of three replicates and analyzed with the PR.ThermControl program provided by NanoTemper Technologies.

### 2.8. Cryo-EM: Grid Preparation, Data Collection, Data Processing, and Model Building

The PGM*_Tk_* sample was prepared in phosphate-buffered saline at a final concentration of 2 mg/mL. Holey carbon grids (Quantifoil R 1.2/1.3, copper, mesh 200) were glow discharged at 25 mA for 30 s using the PELCO easiGlow System immediately before sample loading. Plunge freezing was performed using a Vitrobot Mark IV (Thermo Fisher Scientific^TM^, Waltham, MA, USA) under 100% humidity at 4 °C, and 3 μL of the sample was applied on the grid. Screening and data collection were performed with a Talos Arctica G2 (Thermo Fisher Scientific^TM^, Waltham, MA, USA) operated at 200 kV, equipped with an X-FEG electron source, BioQuantum imaging filter (Gatan), and K3 direct electron detector (Gatan). Imaging was performed in counting mode at a nominal magnification of 100,000×, corresponding to a pixel size of 0.81 Å/pixel. Movies were collected at a total dose of 56.6 e^−^/Å^2^ set at a defocus range of 0.8−2.0 μm.

Movies were imported in CryoSPARC [17] for motion correction and CTF estimation. Further, automated particle picking and 2D classification were performed in CryoSPARC [17]. Classes corresponding to PGM*_Tk_* tetramer were selected for downstream processing and to generate an ab initio map. Heterogenous refinement was performed to remove broken particles or other junk particles from the dataset. Particles corresponding to the most well-defined map were used for non-uniform refinement. Three-dimensional classification was performed in PCA mode to separate particles corresponding to different sub-states, and particles corresponding to a well-defined map were selected. Local refinement of the selected particles resulted in a consensus map corresponding to a 2.45 Å resolution, as assessed by Gold Standard Fourier Shell Correlations (FSCs) using the 0.143 criterion.

A monomer model of PGM*_Tk_* (UniProt ID: Q68BJ6) was generated using AlphaFold2 [12] and downloaded from https://alphafold.ebi.ac.uk/entry/Q68BJ6 (accessed on 11 October 2024). Four copies of the PGM*_Tk_* monomer were rigid-body fitted into the maps using the fit-in-map function of the UCSF ChimeraX (ver. 1.9) [18]. Coot 0.9.1 [19] was used for manual real-space refinement and model building. Phenix ver. 1.20.1 [20] was used for automated real-space refinement. The PGM*_Tk_* model was validated using MolProbity within Phenix ver. 1.20.1 [20].

### 2.9. Structure Deposition

Atomic coordinates and the cryo-EM map were deposited in the Protein Data Bank (PDB ID: 9DU5). The map was also deposited in the Electron Microscopy Data Bank (EMDB ID: 47167).

## 3. Results

### 3.1. Oligomeric State of PGM_Tk_

**Figure 1:**
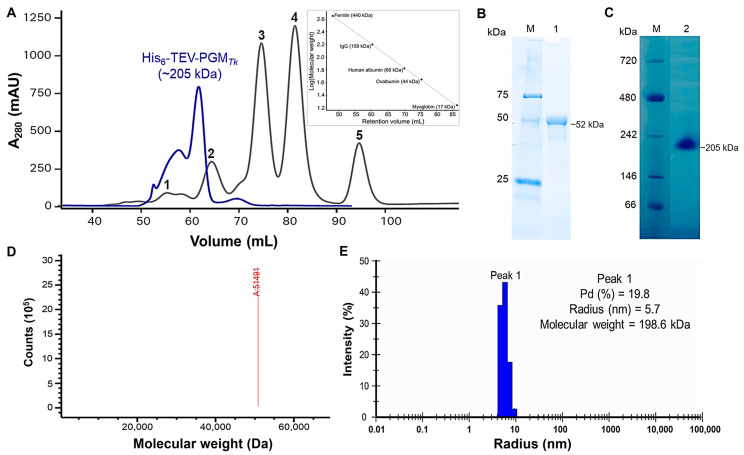
Studies of the oligomerization of PGM*_Tk_*. (**A**) Elution profile of PGM*_Tk_* and standard protein markers using a HiLoad^TM^ 16/60 Superdex^TM^ 200 pg column (Cytiva^TM^, Marlborough, MA, USA). Gray peaks refer to standards: 1, ferritin (440 kDa); 2, IgG (158 kDa); 3, human albumin (66 kDa); 4, ovalbumin (44 kDa); and 5, myoglobin (17 kDa). The highest blue peak corresponds to PGM*_Tk_*. (**B**,**C**) Analysis of PGM*_Tk_* by (**B**) Coomassie brilliant blue stained polyacrylamide gel electrophoresis; denaturing PAGE. (**C**) Non-denaturing-PAGE. Lanes 1 and 2, protein obtained after gel filtration; Lane M, protein marker; SDS-PAGE (Bio-Rad, Hercules, CA, USA_Precision Plus Protein™ Unstained Protein Standards, Cat. #1610363); and Native PAGE (NativeMark™ Unstained Protein Standard, Cat. No. LC0725). (**D**) Mass spectrometry analysis of PGM*_Tk_*. *X*-axis, molecular weight in Daltons; *Y*-axis, intensity (A.U). (**E**) Dynamic light scattering analysis of PGM*_Tk_*. The image of the particle size distribution was taken at a final concentration of 100 µM in 50 mM Tris-HCl (pH 7.0) at 22 °C.

The oligomeric state of PGM*_Tk_* was analyzed with size exclusion chromatography, which confirmed the existence of a tetramer of PGM*_Tk_* with a molecular weight equivalent to ~205 kDa (Figure 1A). Purified PGM*_Tk_* migrated on denaturing and non-denaturing PAGE as a single protein band, with a molecular weight of ~51 kDa (Figure 1B), equivalent to that of a monomer, and ~205 kDa (Figure 1C), equivalent to that of a tetramer, respectively.

The integrity of purified PGM*_Tk_* was analyzed further using electrospray ionization mass spectrometry (ESI-MS). The results revealed a single peak corresponding to a molecular weight of ~51 kDa, which matched the theoretical protomer molecular weight of the recombinant protein (Figure 1D). These results indicated the purified protein is pure and at the expected molecular weight of PGM*_Tk_*.

To assess the presence of aggregates, particle distribution, and their size, DLS was performed. The particle size distribution (Figure 1E) displayed a polydispersity (Pd) value of 19.8%, which is above the 15% threshold, indicating a polydispersity index of the sample. The data also provided approximate values for the hydrodynamic radius (Rh) of individual molecules. The Rh value was 5.7 nm, which corresponded to ~200 kDa. These results confirmed the tetrameric nature of PGM*_Tk_*. These findings are aligned with the results from gel filtration and mass photometry.

**Figure 2:**
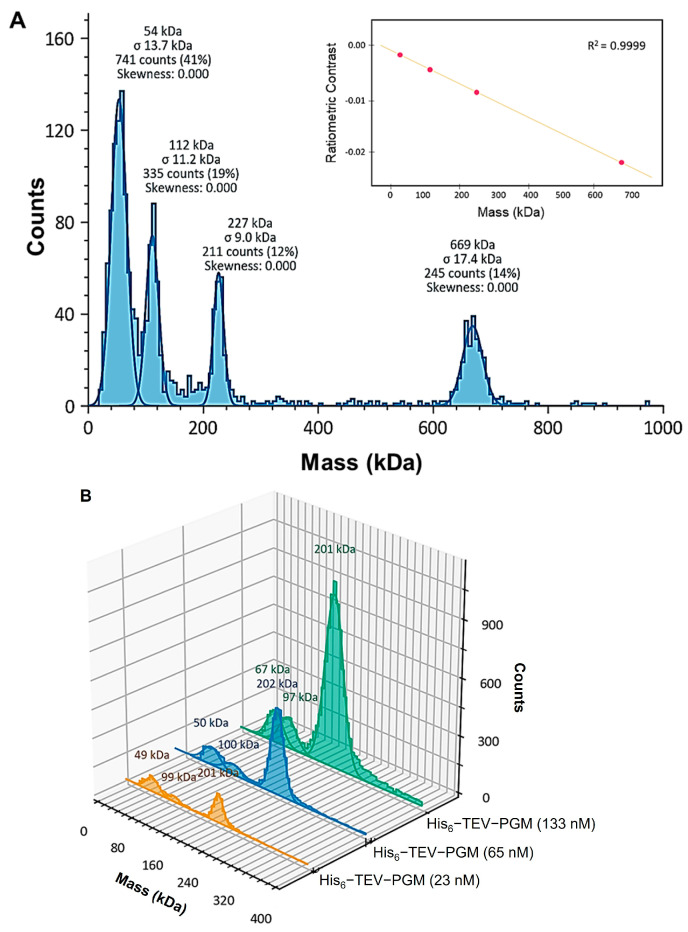
Mass photometry analysis of PGM*_Tk_*. (**A**) The molecular weights were calibrated against the standard proteins with well-defined molecular weights. They include multimeric thyroglobulin (Tg) 335 and 670 kDa and β-amylase (BAM) 56, 112 and 224 kDa, with an error margin of 1.6%. Inset: linear fit of the contrast-to-mass calibration. (**B**) Histograms represent the masses for PGM*_Tk_* at different concentrations. The peaks represent oligomeric states of PGM*_Tk_* at a protein concentration of 133 nM (green), 65 nM (blue), and 23 nM (orange).

Additionally, mass photometry was performed to investigate the molecular weight and oligomeric state of the PGM*_Tk_* complex. The measurements were performed at very low concentrations (23, 65, and 133 nM) to determine if the oligomerization is concentration dependent. The data showed a lesser population of monomers and dimers, whereas the majority of molecules were in a stable tetrameric state (~202 kDa) (Figure 2). The results from multiple experiments confirm the tetrameric nature of PGM*_Tk_*.

### 3.2. Thermal Stability of PGM_Tk_

A differential scanning fluorimetry (DSF) scan was performed to evaluate the thermal and conformational stability of PGM*_Tk_* at pH 7.0, 8.0, and 9.0. The results demonstrate that PGM*_Tk_* remained stable up to at least 85 °C at all the three pH. Moreover, the protein was relatively more stable at alkaline pH, exhibiting a melting temperature of 96 °C at pH 9.0 (Figure S5A).

CD spectrometry analysis was also performed to assess the contents of secondary structure and thermal stability of the protein up to 90 °C. Pronounced negative peaks around 208 and 222 nm indicated a high α-helical content of PGM*_Tk_*. Quantitative analysis performed using DichroWeb [13] shows the prevalence of α-helix (62%), followed by loops and unordered structure (26%) and β-sheets (12%). The CD spectra are quite similar between 40 and 70 °C (Figure S5B). As the temperature reached 80 °C, the negative molar ellipticity decreased, indicating some loss of the α-helical structure. The 90 °C spectrum shows even less α-helical signal, suggesting that PGM*_Tk_* underwent conformational transition from a predominantly α-helical structure to a less structured or random coil state at this temperature.

### 3.3. Cryo-EM Structure of PGM_Tk_

**Figure 3:**
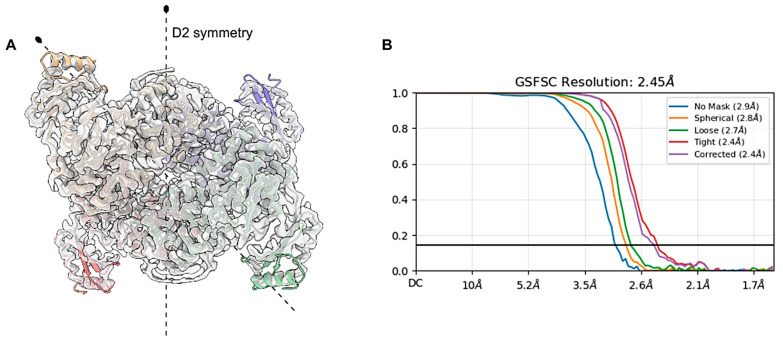
Cryo-EM studies of PGM*_Tk_*. (**A**) A refined model of tetrameric PGM*_Tk_* fitted into a cryo-EM map in D2 symmetry. (**B**) Gold standard Fourier shell correlation (FSC) curve with a nominal resolution of 2.45 Å at 0.143 FSC.

In this study, we present the first cryo-EM structure of any phosphoglucomutase described so far. The map of PGM*_Tk_* was determined at a nominal resolution of 2.45 Å with D2 symmetry, as shown by the GSFSC plot (FSC = 0.143) (Figure 3B). A tetrameric model of PGM*_Tk_* is refined against the PGM*_Tk_* map depicted in Figure 3A. The cryo-EM data collection, processing, structure refinement and validation statistics are shown in Table 1. The cryo-EM data processing workflow is described in Figure S6.

### 3.4. The Protomer of PGM_Tk_

**Figure 4:**
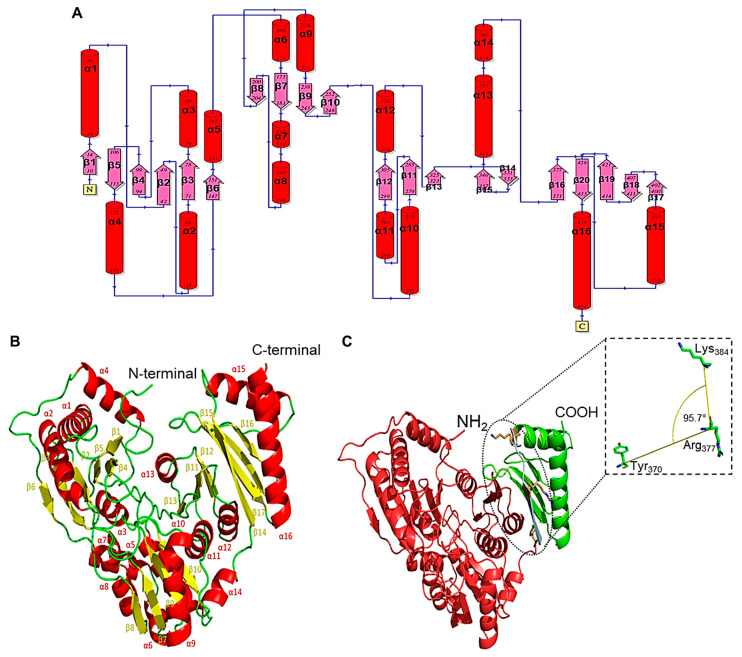
(**A**) A 2D topological diagram showing the three-layer α-β-α sandwich architecture of the protomer of PGM*_Tk_*. The protomer comprises repeating β-loop-α elements. Red cylinders represent α-helices, and pink arrows show β-strands. The diagram was prepared using an online server, PDBSum (https://www.ebi.ac.uk/thornton-srv/databases/pdbsum/ (accessed on 4 January 2025)). (**B**) Secondary structure elements in the protomer of PGM*_Tk_*: α-helix (red), β-sheets (yellow), and loops (green). The labels indicate positions of the respective structural element in the primary structure of the protomer. (**C**) PGM*_Tk_* protomer colored according to domains (N-terminal in red: residues 1–370; C-terminal in green: residues 384–443), whereas the inset shows the angle between the N- and C-terminal domains. Images were prepared using PyMol (ver. 3.0.2).

The protomer of PGM*_Tk_* exhibited a three-layer α-β-α sandwich architecture with a unique α/β topology and arrangement (Figure 4A). It has two distinct regions, N-terminal (domain I, II, and III: residues 1–370) and C-terminal (domain IV: residues 384–443), connected by a helix and a short flexible loop (Figure 4B) comprising Tyr^371^, Gln^371^, Phe^373^, Lys^374^, Thr^375^, Lys^376^, Arg^377^, His^378^, Val^379^, Glu^380^, Gly^381^, Asp^381^, and Arg^383^. Each protomer in the tetrameric PGM*_Tk_* consisted of sixteen α-helices and twenty β-strands. Further, the interdomain angle between the N-terminal and C-terminal domains was calculated using PyMol (ver. 3.0.2). In PGM*_Tk_*, the N- and C-terminal regions adopt a compact, nearly perpendicular orientation, with an interdomain angle of 95.7° (Figure 4C). Moreover, the atomic displacement parameters (ADPs/B-factors) were widely different for different parts of the chain. The N-terminal domain was found to have lower B-factors and showed a rigid symmetry, while the flexible C-terminal region had comparatively larger B-factors.

### 3.5. The Tetramer of PGM_Tk_

**Figure 5:**
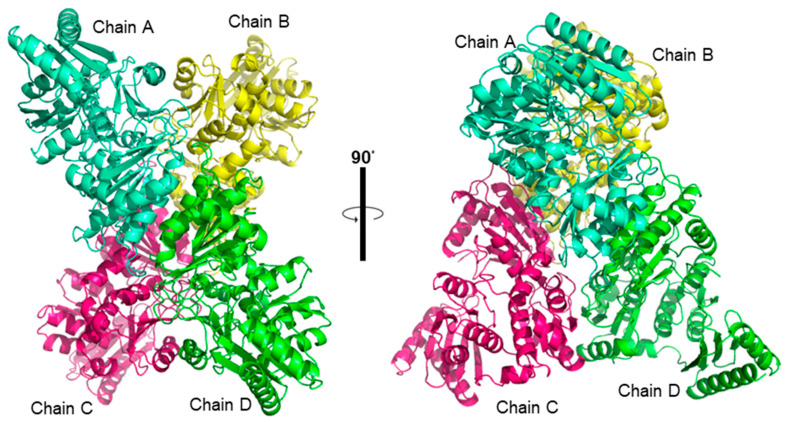
Three-dimensional structure of PGM*_Tk_*. Quaternary structure of PGM*_Tk_* shown from two orientations. Chains A, B, C, and D, are colored in cyan, yellow, magenta, and green, respectively.

The tetrameric structure of PGM*_Tk_* is constituted of two dimers arranged in D2 symmetry (Figure 5). The structural analysis of the protomers in the tetramer display no significant deviations in the conformation. Alignment of 453 residues of chain A to those of chains B, C, and D using GESAMT [21] resulted in root mean square deviation (RMSD) values of 0.18, 0.18, and 0.17 Å, respectively. This suggested the tetrameric assembly of PGM*_Tk_*, underscoring the importance of its symmetrical arrangement.

When the tetrameric structure of PGM*_Tk_* was analyzed for interfaces and subunit organization with PDBePISA (https://www.ebi.ac.uk/pdbe/pisa/; accessed on 14 August 2024) [22], it revealed that the tetramer constitutes a dimer of dimers, chains AD and chains BC pairs. The contact surfaces within these dimers are 1267.6 Å^2^ and 1244.1 Å^2^, respectively. Moreover, the obtained ΔG values of dimers AD and BC are −14.7 and −13.8 kcal/mol, while for dimers AB and CD, these values were −10.2 and −11.1 kcal/mol, respectively. The overall tetrameric assembly exhibited a total surface area of 61,740 Å^2^, comprising 9170 Å^2^ buried and 52,570 Å^2^ solvent accessible areas. Dimer interface interactions included several hydrogen bonds and salt bridges, formed between helix 2 and 3 and loop 1 and 5 of protomers of both the dimers AD (Table S1) and BC (Table S2). In the tetrameric unit, chain A formed a strong interaction with C, while chain B showed a bit weaker interaction with D. The AC (Table S3) and BD (Table S4) assemblies also involved the formation of hydrogen bonds and salt bridges.

### 3.6. Domain Structure Analysis of PGM_Tk_

The CATH (https://www.cathdb.info/ (accessed on 25 August 2024)) [23] and InterProScan (https://www.ebi.ac.uk/interpro/search/sequence/; accessed on 20 July 2024) [24] analysis revealed that PGM*_Tk_* contains four domains that are conserved in hexomutases. Residues 3–138 constitute domain I, residues 156–256 form domain II, residues 261–370 comprise domain III, and residues 384–443 account for domain IV or the C-terminal domain (Figure S7). Domain I includes a conserved region comprising residues 94–105 (^94^GGAVITASHNPP), while domain II harbors three conserved regions consisting of residues 174–194 (^174^RPFVVVDTSNGAGSLTLPYLL), 208–221 (^208^PDGHFPARNPEPNE), and 236–251 (^236^ADFGVAQDGDADRAVF). For the sake of convenience, we consider domains I–III, from residues 3–370, as the N-terminal region. Residues 371 to 383 constitute the hinge region, while residues 384–443 are in the C-terminal region. The first three domains adopt a three-layer α/β/α sandwich architecture, whereas domain IV or the C-terminal domain form a two-layer α/β sandwich topology. Domains I and IV are responsible for the intramolecular phosphotransferase activity [25,26], while domain II is reported to be involved in metal binding to stabilize the overall protein structure [6,27].

### 3.7. Structural Comparison with Thermophilic Phosphohexomutase

**Figure 6:**
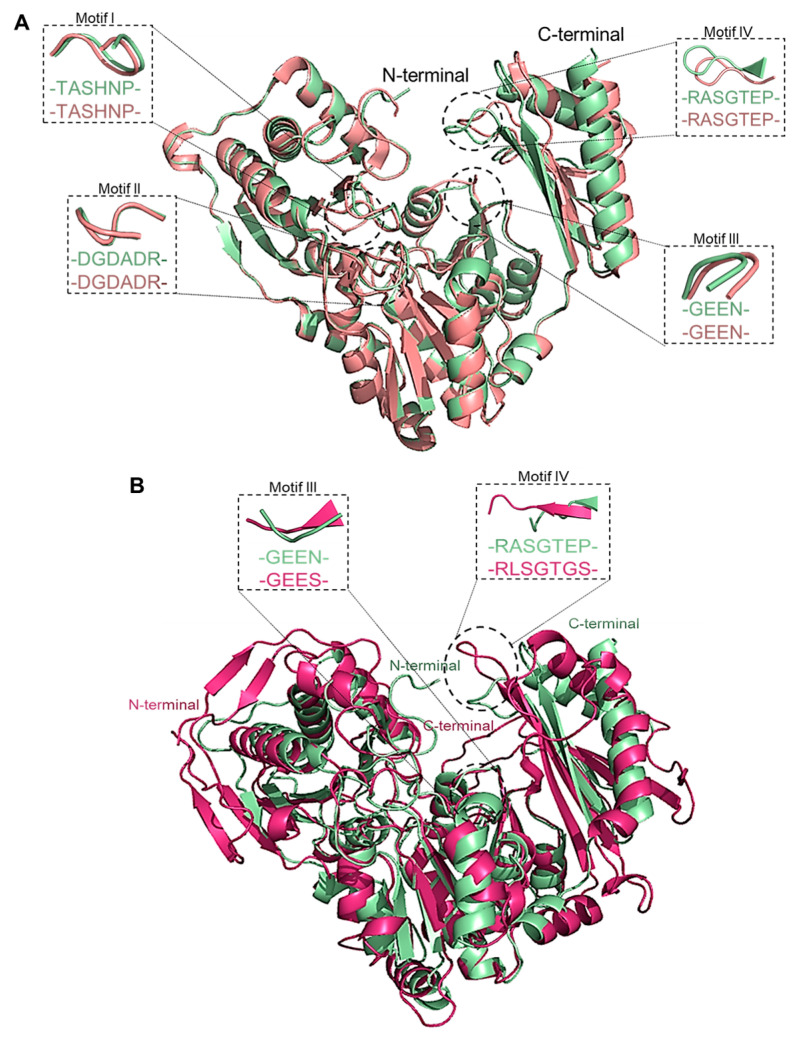
(**A**) Superimposition of the protomers of PGM*_Tk_* (pale green) and PGM*_Ph_* (salmon red) performed by PyMol (ver. 3.0.2). Insets show conserved motifs reported for PGMs. (**B**) Superimposition of the protomers of PGM*_Tk_* (pale green) and PGM*_Oc_* (1LXT: purple-pink) showing the location of modified motifs III and IV in both structures. Alignment was performed using PyMol (ver. 3.0.2).

Analysis using the DALI server [28] showed that PGM*_Tk_* not only exhibits the highest sequence identity but also a high level of structural identity, with a Z-score of 63.9, to that of phosphomannomutase of *Pyrococcus horikoshii* (PGM*_Ph_*) (PDB ID: 1WQA). Superimposition of protomers of PGM*_Tk_* and PGM*_Ph_* using PyMol (ver. 3.0.2) [29] resulted in an RMSD of 1.04 Å (Figure 6A), while independent alignment of N- and C-terminal domains of both structures gave RMSDs of 0.39 and 0.34 Å, respectively. The superposition showed that both proteins possess the same conserved regions and well-aligned residues that show minimal deviations in their spatial positions. Additionally, the comparison of interdomain angles in PGM*_Tk_* (95.7°) and PGM*_Ph_* (98.9°) demonstrated a high degree of conservation in domain arrangement. This observation reinforces the structural similarity and shows functional conservation between the homologs.

### 3.8. Structural Alignment of PGM_Tk_ with Mesophilic Phosphohexomutase

The 3D structure of PGM from rabbit (PGM*_Oc_*) (PDB ID: 1LXT) has been previously determined by X-ray crystallography [30]. The amino acid sequence of this enzyme is 37.1% similar and 22.3% identical to that of PGM*_Tk_*. The structural alignment of PGM*_Tk_* and PGM*_Oc_* performed using PyMol (ver. 3.0.2) showed a higher number of loops and β-sheets in PGM*_Oc_*. An RMSD of 2.04 Å indicates a moderate level of overall structural similarity between both structures. However, when the domains were aligned separately, the N-terminal domain exhibited a lower RMSD of 1.84 Å compared to the C-terminal domain’s RMSD of 2.21 Å, suggesting that the N-terminal domain is more conserved than the C-terminal domain. Additionally, motifs I (-TASHNP-) and II (-DGDADR-) are conserved in both structures while motifs III (PGM*_Tk_*: -GEEN-; PGM*_Oc_*: -GEES-) and IV (PGM*_Tk_*: -RASGTEP-; PGM*_Oc_*: -RLSGTGS-) are different [30,31] (Figure 6B). The greater divergence observed in the C-terminal domain may reflect increased flexibility, variations in ligand-binding regions, or interactions. The interdomain angles, measured as 95.7° for PGM*_Tk_* and 111.5° for PGM*_Oc_*, further support the observed structural differences. This moderate conformational variation is likely attributable to hinge motion between the N- and C-terminal regions. The 95.7° angle suggests a more compact conformation, while the 111.5° angle may represent an intermediate or partially open state.

### 3.9. Structural Comparison with Thermophilic Phosphopentomutase

**Figure 7:**
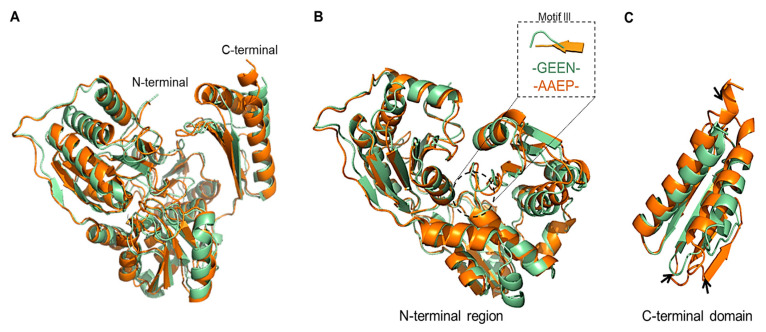
Superimposition of PGM*_Tk_* and PGM*_Ph_*. Performed using PyMol (ver. 3.0.2). (**A**) Complete protomers, (**B**) N-terminal regions (domains I, II, and III), and (**C**) C-terminal domains of PGM*_Tk_* (pale green) and PPM*_Tk_* (orange). Black arrows point towards the misaligned regions.

Structural comparison of PGM*_Tk_* with the crystal structure of phosphopentomutase of *T. kodakarensis* (PPM*_Tk_*) (PDB ID: 9IX8) in GESAMT [21] (CCP4; ver. 8.0.019) showed an alignment of 432 amino acid residues with a sequence similarity of 40.3% and an RMSD of 1.64 Å. Although the apparent conformation of both protomers is quite similar, independent alignment of the N-terminal and C-terminal regions of both structures resulted in an RMSD of 1.05 and 1.32 Å, respectively (Figure 7). The higher RMSD for the C-terminal region may indicate structural flexibility, motion, or functional divergence, and the larger RMSD for the full structures is a sign of relative motions of the domain. Also, the higher flexibility indicates that C-terminal region undergoes conformational change upon substrate-binding. Additionally, the residues of the conserved motif III (-^325^GEEN^328^-; substate binding residues) of PGM*_Tk_* make a loop, while the residues Ala^310^, Ala^311^, and Glu^312^ of the corresponding motif (-^310^AAEP^313^-) in PPM*_Tk_* form a β-sheet, while Pro^313^ forms a turn. This active site architecture may contribute to the catalytic action of both PGM*_Tk_* and PPM*_Tk_* to hexose and pentose substrates, respectively.

### 3.10. Protein–Ligand Interactions

**Figure 8:**
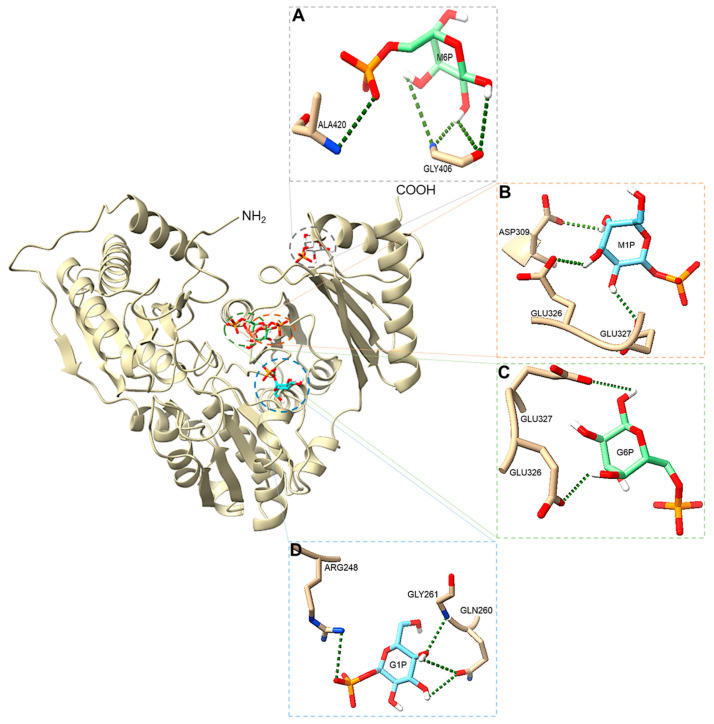
Pictorial representation of interactions between PGM*_Tk_* and hexose substrates, including (**A**) M6P, (**B**) M1P, (**C**) G6P, and (**D**) G1P. Images were prepared with ChimeraX (ver. 1.9).

Multiple sequence alignment confirmed the presence in PGM*_Tk_* of conserved amino acid residues that represent a signature for phosphohexomutases [27] (Figure S8). PGM*_Tk_* was docked with G1P, G6P, mannose 1-phosphate (M1P), and mannose 6-phosphate (M6P); results were viewed using PoseView Online (https://proteins.plus/help/poseview (accessed on 5 November 2024)) [32]. Docking analysis showed the involvement of the sidechains of multiple residues in binding the hexose phosphate substrates, thus highlighting unique substrate binding sites in PGM*_Tk_*. Arg^248^, Gln^260^, and Gly^261^ are involved in binding with G1P, with Arg^248^ residing in the conserved motif II, domain II. Residues from conserved motif III, domain III interact with G6P (Glu^326^ and Glu^327^) and M1P (Asp^309^, Glu^326^, and Glu^327^), while residues Gly^406^ and Ala^420^ from domain IV participate in binding to M6P (Figure 8). The predicted binding affinities for G1P (−5.025 kcal mol^−1^), G6P (−4.987 kcal mol^−1^), M1P (−4.682 kcal mol^−1^), and M6P (−4.565 kcal mol^−1^) indicate that the interaction of PGM*_Tk_* with G1P is the strongest, followed by G6P, M1P, and M6P. Our protein–ligand docking results are in accordance with the reported functions of the domains of hexomutases, where residues from domains I and IV are found to be involved in phosphotransferase activity, and domain II is reported to stabilize the overall protein structure. However, the involvement of multiple residues in binding with different hexose phosphates is unique to PGM*_Tk_*, but that observation should be confirmed by further experimentation based on ligand-bound enzymes.

## 4. Discussion

After a series of reactions, glycogen and starch are converted to G1P, a key intermediate connecting glycogen metabolism with central carbon metabolism. Archaea employ unique enzymes for glycogen metabolism compared to bacteria and eukaryotes. The genome sequence of *T. kodakarensis* harbors four open reading frames annotated as phosphomannomutase. As the size of the genome is very small, 2,088,737 bp with only 2306 genes, having four genes coding for the same function appeared to be very inefficient. Indeed, the gene product of only one of them displayed phosphomannomutase activity. This enzyme exhibited ~1.4-fold higher mutase activity against G6P and was named phosphoglucomutase, PGM*_Tk_* [6]. G6P is often the product of intracellular polysaccharide degradation by various glycan phosphorylases. A common example is the glycolytic reentry of glucose that has been stored as energy in the form of glycogen or trehalose. On the other hand, the substrate of the reverse reaction, G1P, is the precursor of sugar nucleotides that are necessary in the synthesis of various glucose-containing polysaccharides. Therefore, PGM*_Tk_* may also play a biosynthetic role, supplying G1P from G6P that is produced through glycolysis or gluconeogenesis. PGM activity found in *T. kodakaraensis* cells agreed well with the transcription levels of the PGM*_Tk_* gene. Levels of PGM activity were higher in *T. kodakarensis* cells grown on starch than those grown on pyruvate [6]. This indicates a possible role of PGM*_Tk_* in starch degradation, in which glucose 1-phosphate is produced by the function of starch phosphorylases. Another possibility is that this enzyme may be associated with intracellular glycogen synthesis. Putative ADP-glucose synthase (ADP-glucose pyrophosphorylase) and glycogen synthase genes are present in the *T. kodakaraensis* genome. Therefore, there is a possibility that sugars, when abundant, may be stored in *T. kodakarensis* cells in the form of glycogen. Genes homologous to PGM*_Tk_* are also found in the genome sequences of other hyperthermophilic archaea. Three putative PGM/PMM genes are found in *Pyrococcus horikoshii* and they form a gene cluster with the genes encoding putative mannose-1-phosphate guanylyl transferase, mannosyl 3-phosphoglycerate phosphatase, and mannosyl 3-phosphoglycerate synthase. The protein products of the latter two genes have been characterized, and they have clearly shown the expected enzyme activities in the biosynthetic pathway for mannoglycerate [33]. Significant PMM activity of PGM*_Tk_* agrees well with the proposal that genes clustered in the immediate vicinity of the mannosyl 3-phosphoglycerate phosphatase and mannosyl 3-phosphoglycerate synthase genes are involved in mannoglycerate biosynthesis. Given that *T. kodakarensis* is a hyperthermophilic archaeon that thrives in anaerobic environments, PGM*_Tk_* may play a crucial physiological role by effectively regulating cellular glucose homeostasis. This would be particularly useful due to fluctuating nutrient availability in extreme environments.

Here we have reported the biophysical analysis and single particle cryo-EM structure of this novel enzyme. High thermal stability of the protein was assessed using CD spectrometry and differential scanning fluorimetry. CD spectrometry demonstrated a decrease in α-helical content of PGM*_Tk_* with increasing temperature. Mass photometry and dynamic light scattering also verified the oligomerization results of size exclusion chromatography, which showed that PGM*_Tk_* existed as a tetramer. Further computational analysis showed that the structure is made up of dimers of dimers that interact through H-bonds and salt bridges. Substrate binding to domain III may help in the conformational orientation of the active site for phosphotransferase activity.

Computational analysis demonstrated the involvement of residues Arg^248^, Gln^260^, and Gly^261^, Asp^309^, Glu^326^, Glu^327^, Gly^406^, and Ala^420^ from domains II and III in binding to the substrate, making these residues potentially responsible for the phosphomutase activity of PGM*_Tk_*. Additionally, structural comparison with the reported crystal structure of the closest homolog, PGM*_Ph_*, indicated the involvement of residues Ser^101^ (from the conserved motif I and domain I) and Asp^243^, Asp^245^, and Asp^247^ (from the conserved motif II and domains II) in binding to the substrate, which help in the conformational orientation of the active site suitable for phosphotransferase activity at elevated temperatures.

A comparison PGM*_Tk_* with PGM*_Ph_* and a commercially available mesophilic ortholog (PGM*_Os_*) demonstrates the presence of conserved regions, hence linking to the evolution of PGM*_Tk_* over the time in hyperthermophilic and mesophilic organisms. Additionally, PGM*_Tk_* showed a configuration of secondary structural elements that is unique to the reported PGMs. Additionally, PGM*_Tk_* and PPM*_Tk_* alignment results demonstrated that the proteins with high sequence similarity may have different functions or can catalyze different substrates.

The alignment of PGM*_Tk_* structures predicted by AlphaFold2 and experimentally obtained using cryo-EM showed an RMSD of only 0.41 Å. There is no doubt that AlphaFold2 has revolutionized the field by rapidly and efficiently predicting protein structures using the currently available database of experimentally determined protein structures. However, even a minor discrepancy in defining the flexible regions could cause misinterpretation of the interactions with the substrates and thus influence analysis of enzyme activity or stability. Thus, experimentally determined protein structures remain the gold standard, although their determination might sometimes be challenging and time-consuming. Ideally, these approaches complement each other, with AlphaFold guiding initial analyses and experimental structures providing definitive validation.

## 5. Conclusions

This study characterizes the unique phosphoglucomutase from *T. kodakarensis* (PGM*_Tk_*) with its tetrameric cryo-EM structure. We describe key residues involved in substrate binding. Structural comparisons highlight evolutionary adaptations and distinct features that set PGM*_Tk_* apart from other homologs, such as PGM*_Ph_* and PGM*_Oc_*. The findings underscore the critical role of experimental structural determination in complementing computational predictions and provide a foundation for future research into the dynamics and functions of hyperthermophilic enzymes.

## Figures and Tables

**Table 1:** Cryo-EM data collection and refinement statistics for PGM*_Tk_*.

**Data Collection**	
Microscope	Talos Arctica
Voltage (kV)	200
Camera	Gatan K3
Camera mode	Counting
Magnification	100,000
Data acquisition software	EPU
Exposure navigation	Image shift
Total electron exposure (e^−^/Å^2^)	56.6
Exposure rate (e^−^/pixel/sec)	14.85
Frame length (ms)	62.5
Number of frames	40
Pixel size (Å/pixel)	0.81
Defocus range at scope (µm)	0.8–2
Measured defocus range (µm)	0.1–3.9
Micrographs collected (no.)	8168
**Data analysis**	
Image processing package	CryoSparc
Total extracted picks (no.)	3.67 M
Particles used for 3D (no.)	1.5 M
Final Particles (no.)	645, 208
Symmetry	D2
Resolution (Å)	
FSC 0.143 (unmasked/masked)	2.9/2.4
Local resolution range (Å)	1.7/33.2
Map sharpening B-factor (Å^2^)	78.92
**Model composition**	
Protein residues	1806
Ligands	0
**Model Refinement**	
Refinement package	Phenix
MapCC (volume/mask)	0.84/0.85
B-factors (Å^2^)	
Protein residues	109.09
Ligands	N/A
R.m.s. deviations	
Bond lengths (Å)	0.003
Bond angles (◦)	0.493
**Validation**	
Map-to-model, FSC 0.5	2.71
Ramachandran plot	
Favored (%)	98.16
Allowed (%)	1.84
Outliers (%)	0
MolProbity score	1.47
Clashscore	6.63
Poor rotamers (%)	1.37
C-beta deviations	0
CaBLAM outliers (%)	1.68

## Data Availability

The data presented in this study are openly available in PDB reference number 9DU5 [Protein Data Bank] [https://www.rcsb.org/structure/unreleased/9DU5, accessed on 2 October 2024] [9DU5].

## Supplementary Materials

The following supporting information can be downloaded at https://www.mdpi.com/article/10.3390/biom15030319/s1, Figure S1. Pictorial representation of Quick-change PCR steps for constructing the expression vector, pET-His_6_-TEV-Tk1108. (A) PCR profile for step 1: primer annealing and extension, (B) PCR profile for step 2: construct extension, (C) agarose gel showing; HR: Invitrogen E-Gel™ 96 High Range DNA Ladder (Product No. MAN0001082); LR: Invitrogen E-Gel™ Low Range Quantitative DNA Ladder (Product No. MAN0001085); Lane 1: purified PCR product (6927 bp). Figure S2. Agarose gel (Invitrogen E-Gel™ EX Agarose Gels, Cat. No. G401001) analysis for the restriction digestion of the construct pET-His_6_-TEV-Tk1108 with BamHI and NdeI. HR: Invitrogen E-Gel™ 96 High Range DNA Ladder (Product No. MAN0001082); LR: Invitrogen E-Gel™ Low Range Quantitative DNA Ladder (Product No. MAN0001085); Lane 1 shows the integral construct (6927 bp); and Lane 2 shows the bands of size 1413 bp and 5514 bp obtained after restriction digestion. Figure S3. The computer-generated electropherogram shows the nucleotide sequencing for His_6_-TEV-Tk1108. The expression construct was subjected to Sanger sequencing to confirm the product of Quick-change PCR. Figure S4. SDS-PAGE analysis of the expression of His_6_-TEV-Tk1108 in *E. coli* strains; BL21-CodonPlus^®^(DE3)-RIPL and Rosetta^TM^ 2(DE3) pLysS Singles^TM^. Protein production was carried out at 18 °C using 1 mM IPTG (final concentration) as an inducer. Lane M displays protein marker (BioRad_Precision Plus Protein™ Unstained Protein Standards, Cat. #1610363); Lane S and P show soluble fractions and inclusion bodies, respectively. Figure S5. Thermal stability analysis of PGM_Tk_. (A) Differential scanning fluorimetry analysis. Protein samples were analyzed in Tris-HCl buffer, pH 7.0 (green), 8.0 (blue), and 9.0 (orange). (B) Analysis of the secondary structure elements of PGM*_Tk_* by CD spectrometry. CD spectrum is shown after subtraction of the data of 5 mM Tris-HCl buffer (pH 8.0). CD scan was performed from 260 to 200 nm, with a final protein concentration of 0.3 mg/mL at a temperature range of 40–90 °C. Figure S6. Cryo-EM studies of PGM*_Tk_*. Image shows Single-particle cryo-EM processing workflow and reconstructions of PGM*_Tk_*. Figure S7. Graphical representation of domains of PGM*_Tk_* (455 residues). The backbone of the protein is shown in grey, while domains I, II, III, and IV are colored blue, red, yellow, and cyan, respectively. Figure S8. Multiple sequence alignment (MSA) of PGM*_Tk_* (PDB ID: 9DU5) with PGM*_Ph_* (PDB ID: 1WQA), PGM*_Oc_* (PDB ID: 1LXT), and PPM*_Tk_* (PDB ID: 9IX8) accomplished using Clustal Omega (https://www.ebi.ac.uk/jdispatcher/msa/clustalo (accessed on 30 December 2024)). Sequence alignment shows the presence of four signature motifs for PGMs. Motifs I and II are conserved in all four aligned sequences, while motifs III and IV are conserved in PGM*_Tk_* and PGM*_Ph_* only. Symbol ‘*’ (asterisk) shows identical residues; a ‘.’ (period) specifies residues with weekly similar properties; a ‘:’ (colon) symbolizes residues with strongly alike properties; and a ‘-’ (hyphen) shows the gap. Table S1. Bonds in the interfaces between the principal dimer, AD, of PGM*_Tk_*. Table S2. Bonds in the interfaces between the principal dimer, BC, of PGM*_Tk_*. Table S3. Hydrogen bonds and salt bridges between protomers A and C of PGM*_Tk_*. Table S4. Hydrogen bonds and salt bridges between protomers B and D of PGM*_Tk_*.
