# Supplementary material for: A Bifunctional Phosphoglucomutase/Phosphomannomutase from Thermococcus kodakarensis: Biophysical Analysis and Cryo-EM Structure
Source: Biomolecules. 2025 Feb 21;15(3):319. doi: 10.3390/biom15030319 (PMC11940775; doi:10.3390/biom15030319)
Supplement: Supplementary file 1 [file biomolecules-15-00319-s001.zip › biomolecules-3453129-supplementary.pdf]

biomolecules

MDPI

Article

A Bifunctional Phosphoglucomutase/Phosphomannomutase from *Thermococcus kodakarensis*: Biophysical Analysis and Cryo-EM Structure

Zahra Naz [1,2,†], Ishan Rathore [2,†], Muhammad Saleem [1], Moazur Rahman [1], Alexander Wlodawer [2,*] and Naeem Rashid [1,*]

1 School of Biological Sciences, University of the Punjab, Lahore 54590, Pakistan
2 Center for Structural Biology, National Cancer Institute, National Institutes of Health, Frederick, MD 21702, USA
* Correspondence: wlodawer@nih.gov (A.W.); naeem.ff.sbs@pu.edu.pk or naeemrashid37@hotmail.com (N.R.)
† These authors contributed equally to this work.

 https://doi.org/10.3390/biom15030319

1. Construction of the Expression Vector, pET-His$_6$-TEV-Tk1108, Using Quick-Change PCR

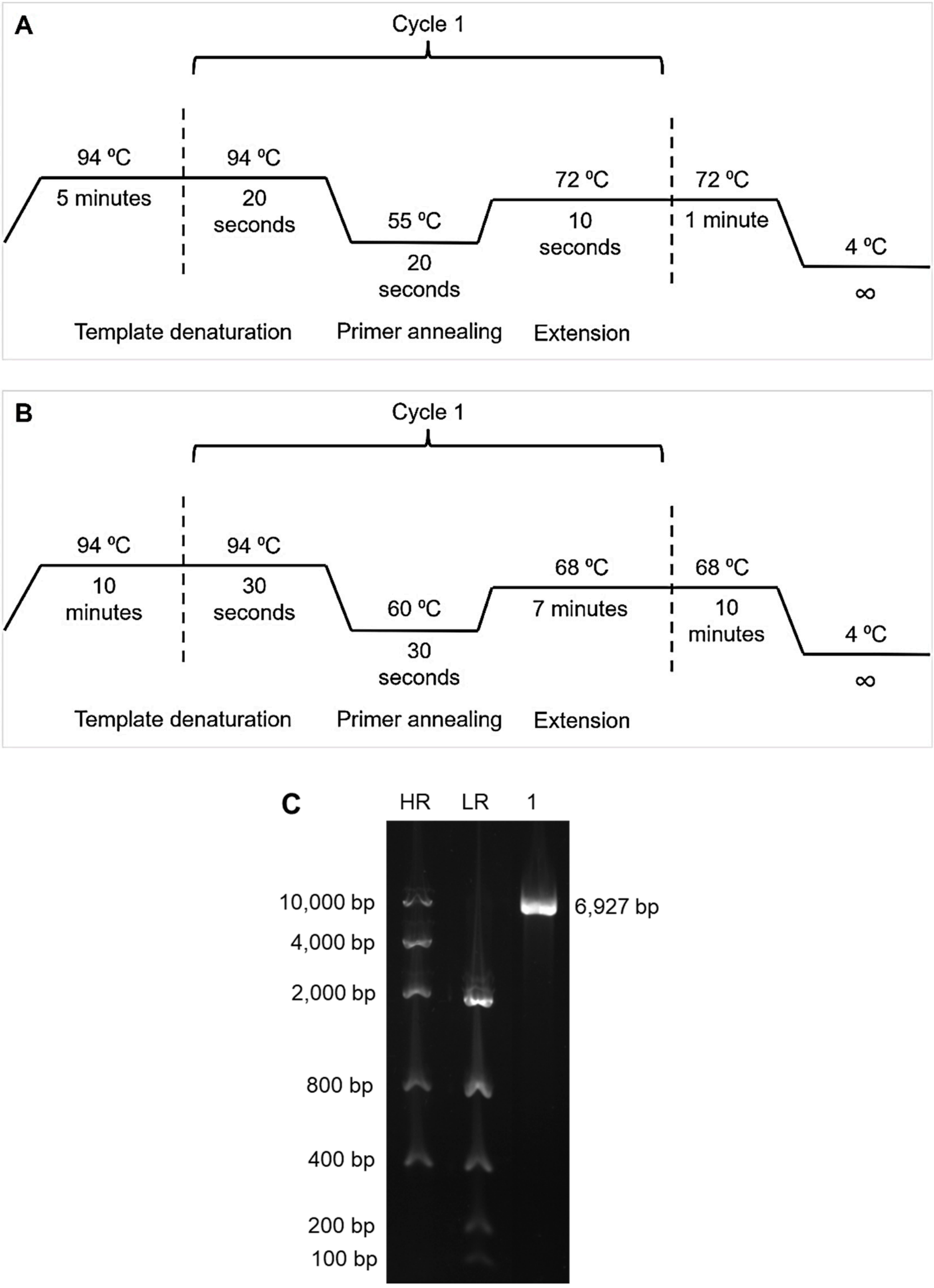

Figure S1. Pictorial representation of Quick-change PCR steps for constructing the expression vector, pET-His$_6$-TEV-Tk1108. (**A**) PCR profile for step 1: primer annealing and extension, (**B**) PCR profile for step 2: construct extension, (**C**) agarose gel showing; HR: Invitrogen E-Gel™ 96 High Range DNA Ladder (Product No. MAN0001082); LR: Invitrogen E-Gel™ Low Range Quantitative DNA Ladder (Product No. MAN0001085); Lane 1: purified PCR product (6,927 bp).

2. Restriction Digestion Analysis of the Expression Vector, pET-21a(+)-His$_6$-TEV-Tk1108

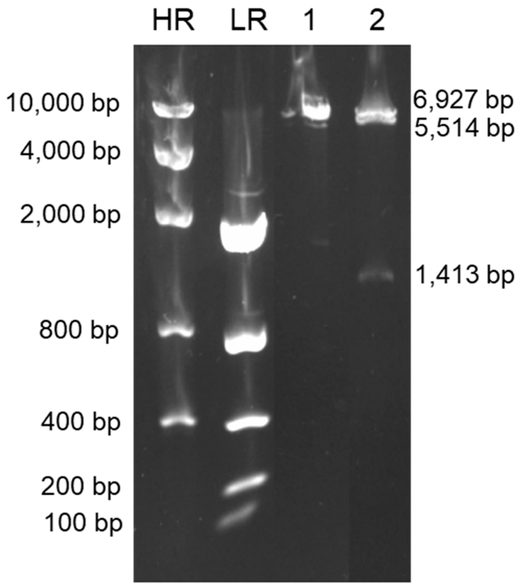

Figure S2. Agarose gel (Invitrogen E-Gel™ EX Agarose Gels, Cat. No. G401001) analysis for the restriction digestion of the construct pET-21a(+)-His$_6$-TEV-Tk1108 with *Bam*HI and *Nde*I. HR: Invitrogen E-Gel™ 96 High Range DNA Ladder (Product No. MAN0001082); LR: Invitrogen E-Gel™ Low Range Quantitative DNA Ladder (Product No. MAN0001085); Lane 1 shows the integral construct (6,927 bp); and Lane 2 shows the bands of size 1,413 bp, and 5,514 bp, obtained after restriction digestion.

3. Sequencing Electropherogram for His$_6$-TEV-Tk1108

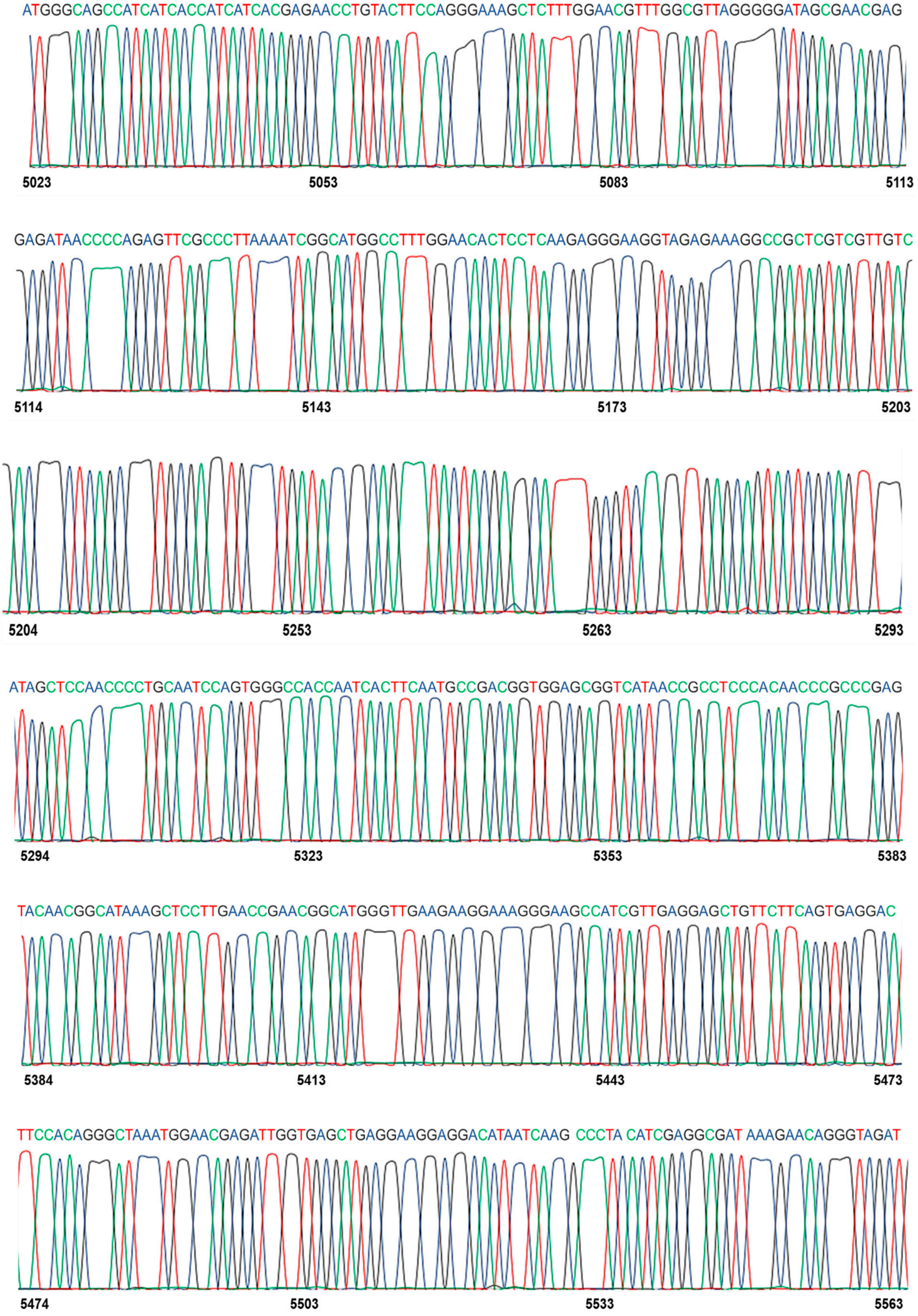

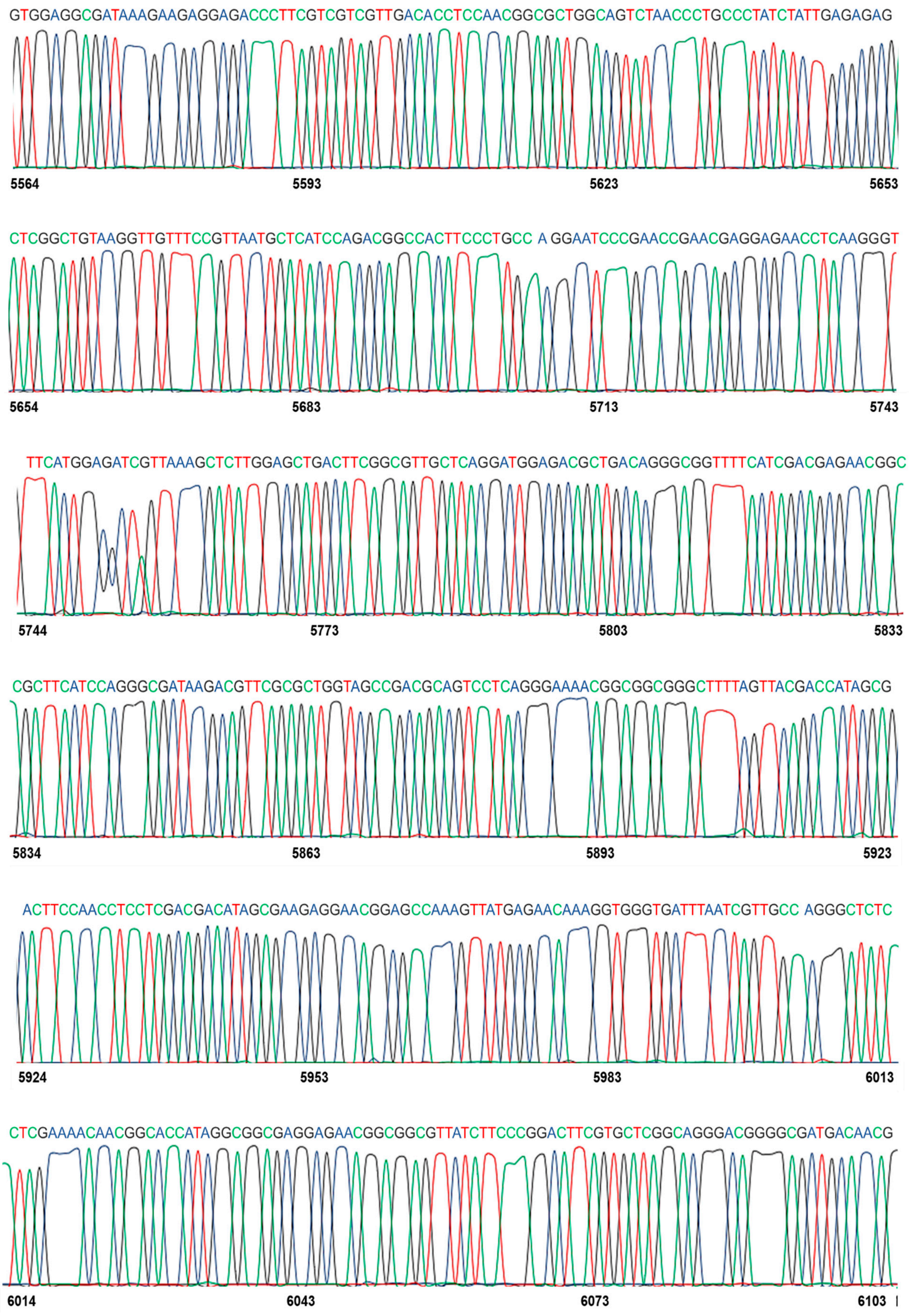

GTGGAGGCGATAAAGAAGAGGAGACCCTTCGTCGTCGTTGACACCTCCAACGGCGCTGGCAGTCTAACCCTGCCCTATCTATTGAGAGAG
5564
5593
5623
5653
CTCGGCTGTAAGGTTGTTTCCGTTAATGCTCATCCAGACGGCCACTTCCCTGCC A GGAATCCCGAACCGAACGAGGAGAACCTCAAGGGT
5654
5683
5713
5743
TTCATGGAGATCGTTAAAGCTCTTGGAGCTGACTTCGGCGTTGCTCAGGATGGAGACGCTGACAGGGCGGTTTTCATCGACGAGAACGGC
5744
5773
5803
5833
CGCTTCATCCAGGGCGATAAGACGTTCGCGCTGGTAGCCGACGCAGTCCTCAGGGAAAACGGCGGCGGGCTTTTAGTTACGACCATAGCG
5834
5863
5893
5923
ACTTCCAACCTCCTCGACGACATAGCGAAGAGGAACGGAGCCAAAGTTATGAGAACAAAGGTGGGTGATTTAATCGTTGCC AGGGCTCTC
5924
5953
5983
6013
CTCGAAAACAACGGCACCATAGGCGGCGAGGAGAACGGCGGCGTTATCTTCCCGGACTTCGTGCTCGGCAGGGACGGGGCGATGACAACG
6014
6043
6073
6103

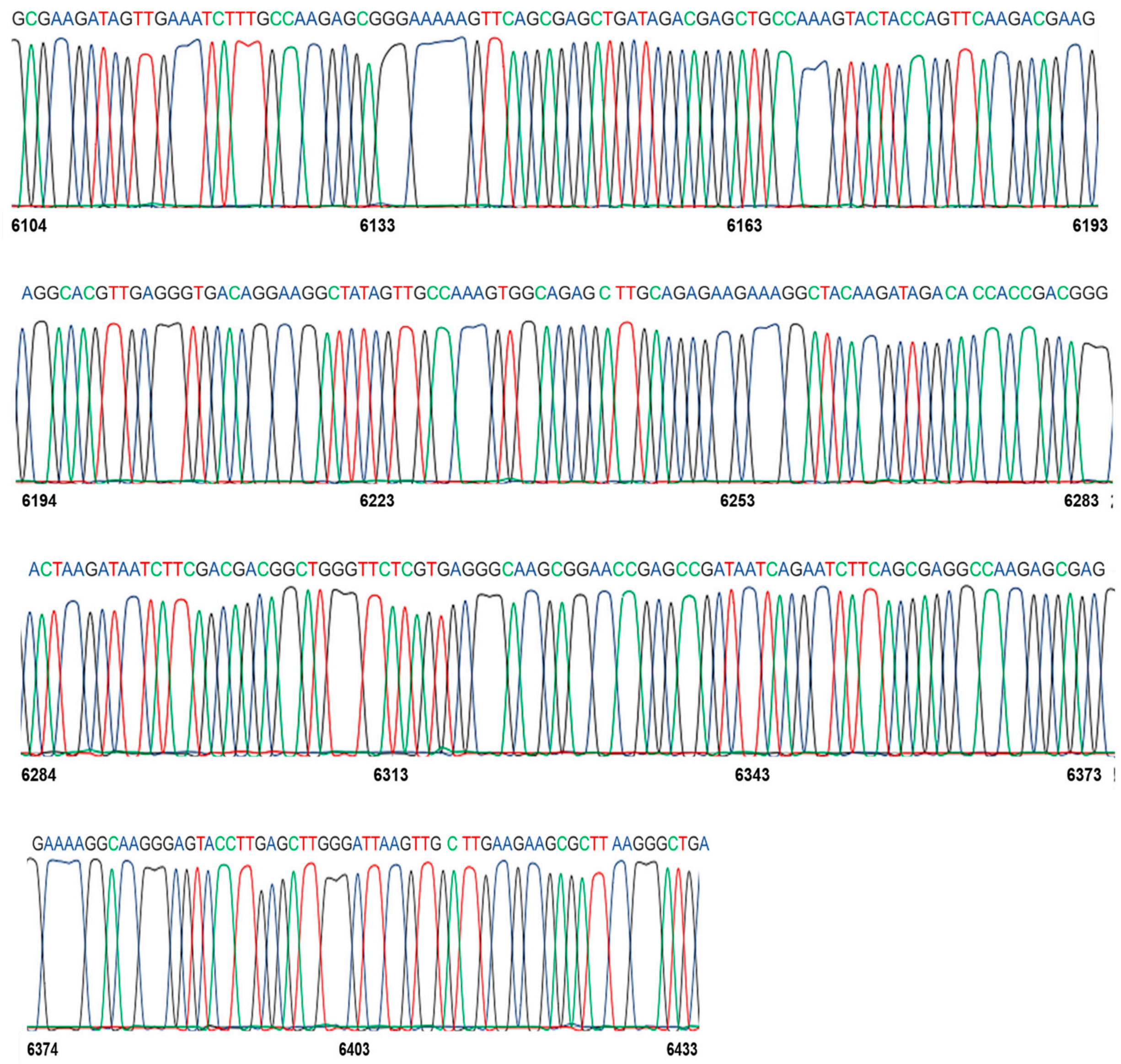

Figure S3. The computer-generated electropherogram shows the nucleotide sequencing for His_6-TEV-Tk1108. The expression construct was subjected to Sanger sequencing to confirm the product of Quick-change PCR.

4. SDS-PAGE Analysis of the Expression of His$_6$-TEV-Tk1108 in *E. coli* Strains

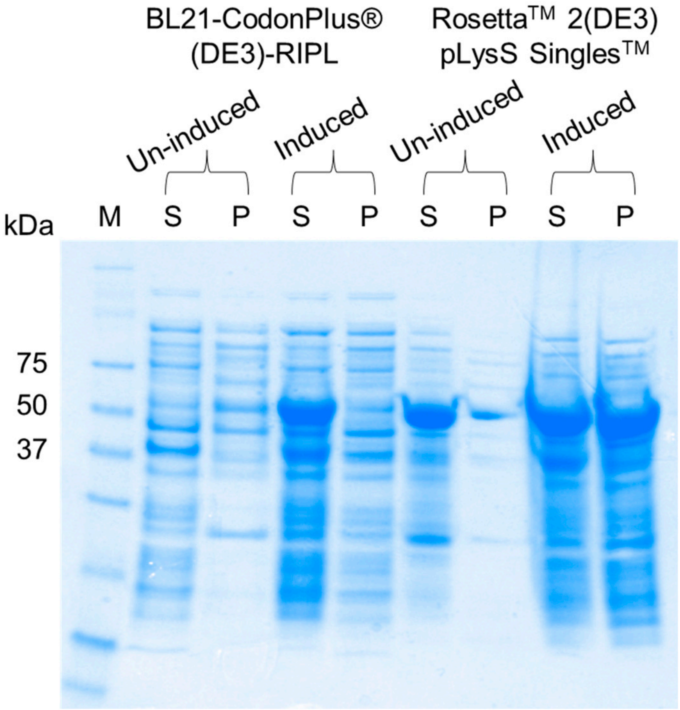

Figure S4. SDS-PAGE analysis of the expression of His$_6$-TEV-Tk1108 in *E. coli* strains; BL21-CodonPlus®(DE3)-RIPL, and Rosetta™ 2(DE3) pLysS Singles™. Protein production was done at 18 °C, using 1 mM IPTG (final concentration) as an inducer. Lane M displays protein marker (BioRad_Precision Plus Protein™ Unstained Protein Standards, Cat. #1610363); Lane S and P show soluble fractions, and inclusion bodies, respectively.

5. Thermal Stability Analysis of PGM$_{Tk}$

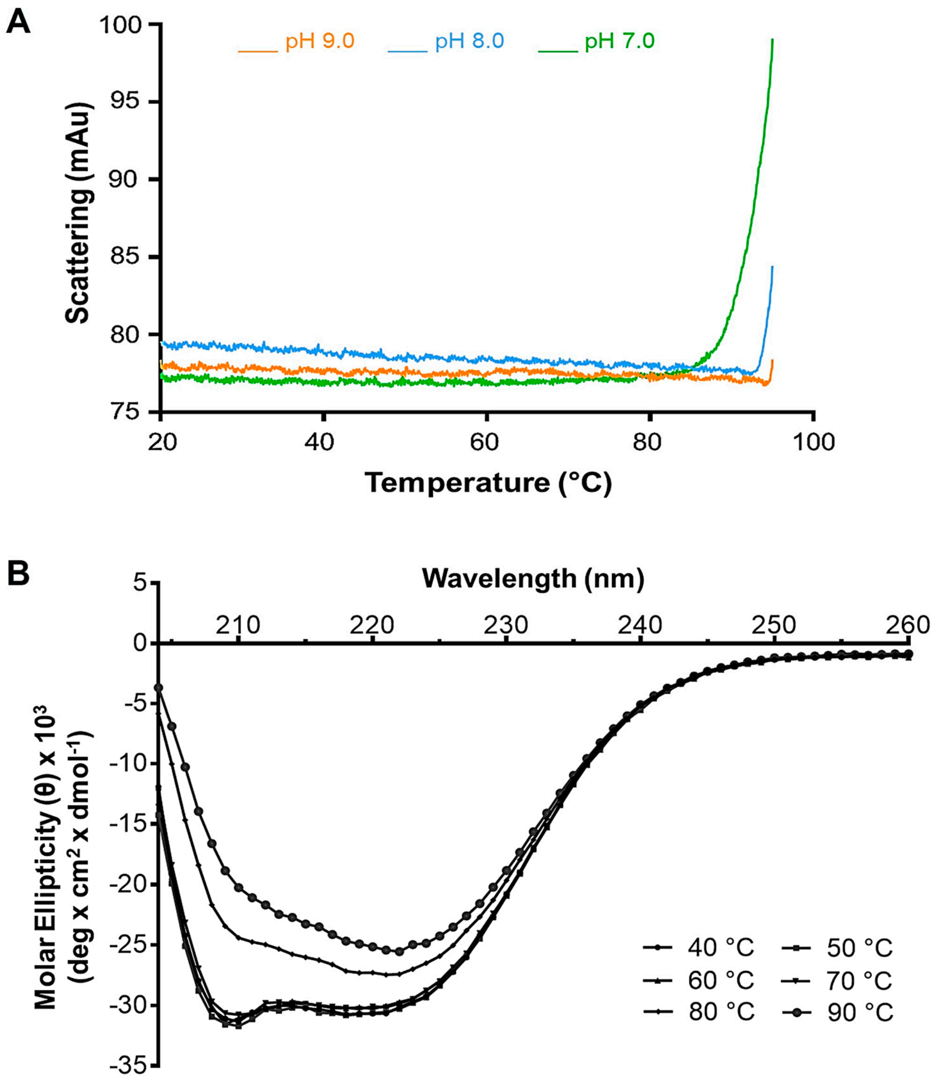

Figure S5. Thermal stability analysis of PGM$_{Tk}$. (**A**) Differential scanning fluorimetry analysis. Protein samples were analyzed in Tris-HCl buffer pH 7.0 (green), 8.0 (blue), and 9.0 (orange). (**B**) Analysis of the secondary structure elements of PGM$_{Tk}$ by CD spectrometry. CD spectrum is shown after subtraction of the data of 5 mM Tris-HCl buffer (pH 8.0). CD scan was performed from 260–200 nm with a final protein concentration of 0.3 mg/mL at a temperature range of 40–90 °C.

6. Workflow for cryo-EM of PGM*Tk*

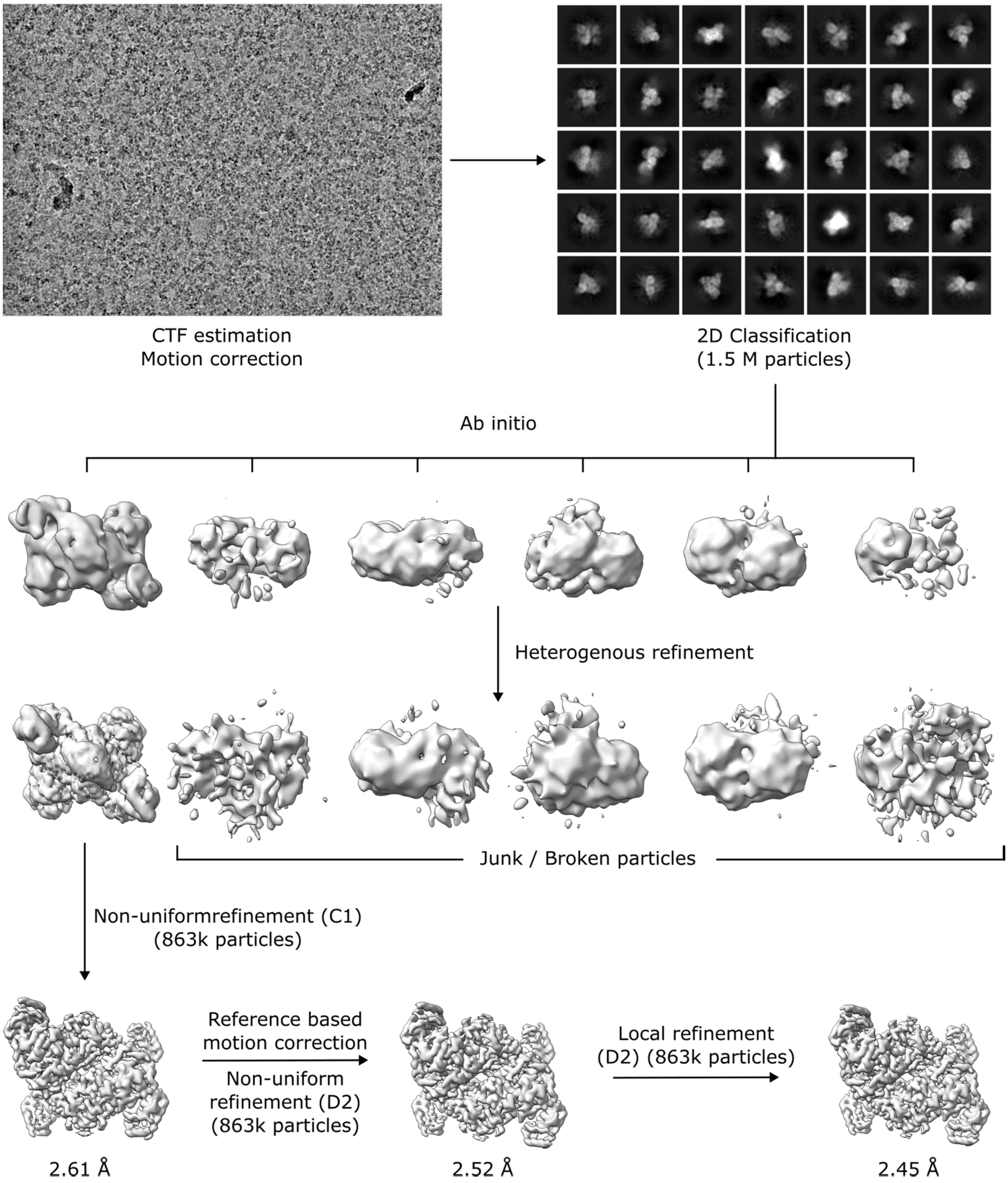

Figure S6. Cryo-EM studies of PGM*Tk*. Image shows Single-particle cryo-EM processing workflow and reconstructions of PGM*Tk*.

7. Domains of Phosphoglucomutase

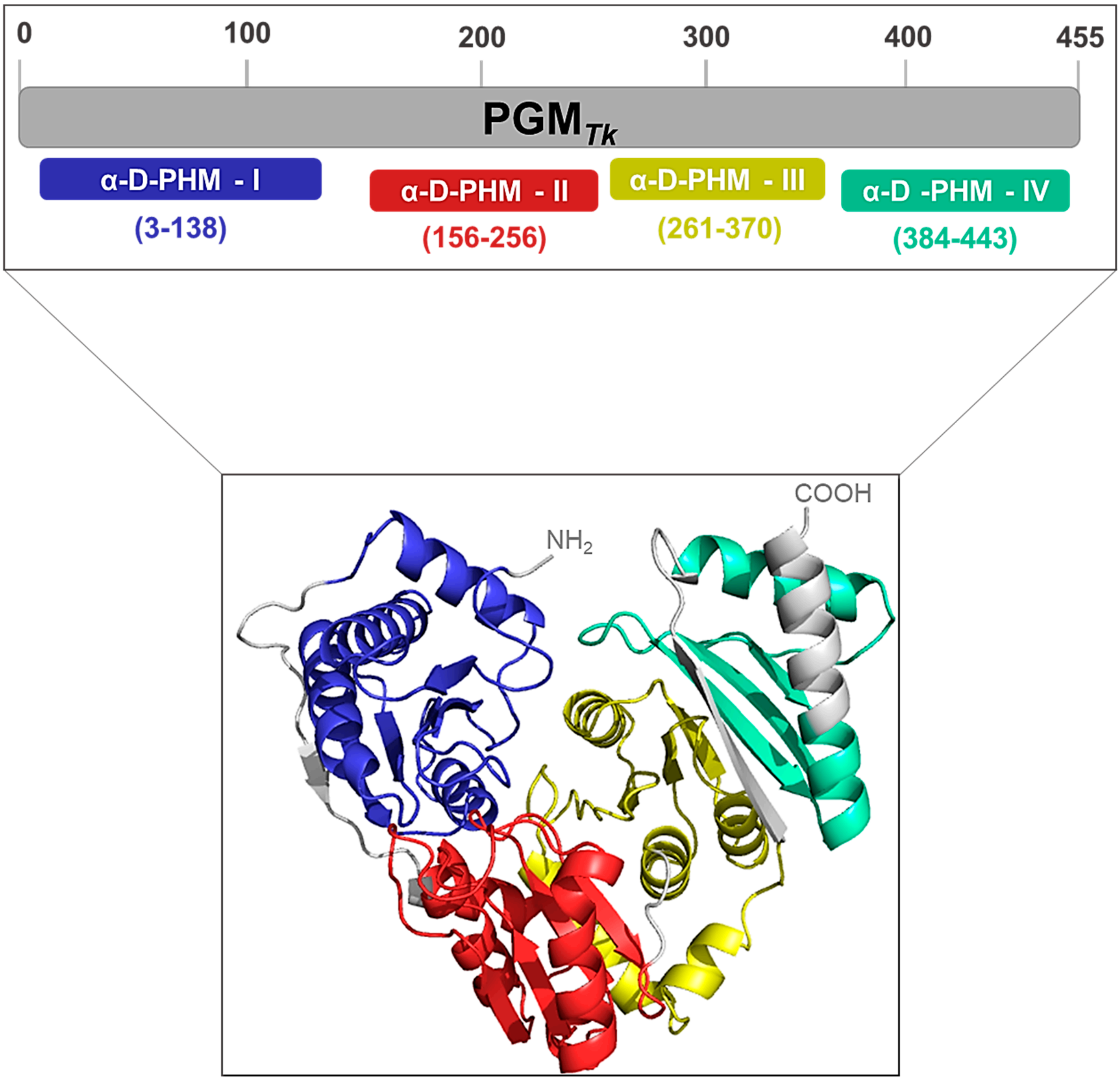

Figure S7. Graphical representation of domains of PGM$_{Tk}$ (455 residues). The backbone of the protein is shown in grey, while domains I, II, III and IV are colored blue, red, yellow, and cyan, respectively.

8. Multiple Sequence Alignment of PGM*Tk* with homologs

```
9DU5            -----------MGKLFGTFGVRGIANEE-ITPEFALKIGMAFGTLLKREGRERPLVVVGR  48
1WQA            -----------MGKLFGTFGVRGIANEK-ITPEFAMKIGMAFGTLLKREGRKKPLVVVGR  48
1LXT            VKIVTVKTKAYPDQKPGTSGLRKRVKVFQSSTNYAENFIQSIISTVEPAQRQEATLVVGG  60
9IX8            -------------RLFGTAGIRGTLWEK-VTPELAMKVGMAVGTYKS------GKALVGR  40
                             :  ** *:*        : : * :.  :. :  .         :**

9DU5            DTRVSGEMLKDALISGLL--STG-CDVIDVGIAPTPAIQWATNHFNADGGAVITASHNPP  105
1WQA            DTRVSGEMLKEALISGLL--SVG-CDVIDVGIAPTPAVQWATKHFNADGGAVITASHNPP  105
1LXT            DGRFYMKEAIQLIVRIAAANGIGRLVIGQNGILSTPAVSCIIRKIKAIGGIILTASHNPG  120
9IX8            DGRTSSVMLKNAMISGLL--STG-MEVLDADLIPTPALAWGTRKL-ADAGVMITASHNPP  96
                * *       : ::      . *   : : .:  ***:    .:: * .* ::******
                                                                   Motif I

9DU5            EYNGIK---LLEPNGMGLKKEREAIVEELFFS----------E------------DFHRA  140
1WQA            EYNGIK---LLEPNGMGLKKEREAIVEELFFK----------E------------DFDRA  140
1LXT            GPNGDFGIKFNISNGGP---APEAITDKIFQISKTIEEYAICPDLKVDLGVLGKQQFDLE  177
9IX8            TDNGVK---VFNGDGTEFYVEQERGLEEIIFS----------G------------NFRKA  131
                  **     .   :*       *   ::::                         :*

9DU5            KWNEIGELRKEDIIKPYIEAIKNRVDVEAIKK-----RRPFVVVDTSNG-AGSLTLPYLL  194
1WQA            KWYEIGEVRREDIIKPYIEAIKSKVDVEAIKK-----RKPFVVVDTSNG-AGSLTLPYLL  194
1LXT            NKFKPFTVEIVDSVEAYATMLRNIFDFNALKELLSGPNRLKIRIDAMHGVVGPYVKKILC  237
9IX8            RWDEIKPVRNVEVIPDYINAVLDFVGHET---------NLKVLYDGANG-AGSLVAPYLL  181
                .  :   :.  : :  *   : . .. ::         .  :  *  :* .*  .   *

9DU5            RELGCKV-VSVNAHPDGHFPARNPEPNEENLKGFMEIVKALGADFGVAQDGDADRAVFID  253
1WQA            RELGCKV-ITVNAQPDGYFPARNPEPNEENLKEFMEIVKALGADFGVAQDGDADRAVFID  253
1LXT            EELGAPANSAVNCVPLEDFGGHHPDPNLTYAADLVETMKSGEHDFGAAFDGDGDRNMILG  297
9IX8            REMGAKV-LSVNAHVDGHFPGRKPEPRYENIAYLGKLVRELGVDLAIAQDGDADRIAVFD  240
                .*:*. .  :**.     * .::*:*.      : : ::    *:. * *** **  .:.
                                                                Motif II

9DU5            ENGRFIQGDKTFALVAD-----AVLRENGGGLLVTTIATSNLLDDIAKRNGAKVMRTKVG  308
1WQA            ENGRFIQGDKTFALVAD-----AVLKEKGGGLLVTTVATSNLLDDIAKKHGAKVMRTKVG  308
1LXT            KHGFFVNPSDSVAVIAANIFSIPYFQQTGVRGFARSMPTSGALDRVANATKIALYETPTG  357
9IX8            EKGNYVDEDTVIALFAK-----LYVEEHGGGTVVVSIDTGSRIDAVVERAGGRVVRIPLG  295
                ::* ::: .  .*:.*        ..: *   .. :: *.. :* :.:     : .   *

9DU5            DLIVARALLENNGTIGGEENGGVIFPDFVLGRDGAMTTAKIVEIFAKSGKKFSELIDELP  368
1WQA            DLIVARALYENNGTIGGEENGGVIFPEHVLGRDGAMTVAKVVEIFAKSGKKFSELIDELP  368
1LXT            WKFFGNLMDASKLSLCGEESFGTG-SDHIREKDGLWAVLAWLSILATRKQSVEDILKDHW  416
9IX8            QPHDGIKRYK---AIFAAEPWKLVHPKFGPWIDPFVTMGLLIK-LIDENGPLSELVKEIP  351
                    .        ::   *       ..    *   :    :. :      ..:::.:
                                Motif III

9DU5            --------KYYQFKTKRHVEGD------RKAIVAKVAELAEKKG-----Y----------  399
1WQA            --------KYYQIKTKRHVEGD------RHAIVNKVAEMARERG-----Y----------  399
1LXT            HKFGRNFFTRYDYEE-VEAEGATKMMKDLEALMFDRSFVGKQFSANDKVYTVEKADNFEY  475
9IX8            --------TYYLKKANVLCPDE------YKAEVVRRAAEEVERKLSSEIK----------  387
                        . *  :      .       .*  :   :    :

9DU5            ------KIDTTDGTKI-IFDDGWVLVRASGTEP---IIRIFSEAKSEEKARE---YLELG  446
1WQA            ------TVDTTDGAKI-IFEDGWVLVRASGTEP---IIRIFSEAKSKEKAQE---YLNLG  446
1LXT            HDPVDGSVSKNQGLRLIFADGSRIIFRLSGTGSAGATIRLYIDSYEKDNAKINQDPQVML  535
9IX8            ------EVLTISGFRIALNDGSWILIRPSGTEP---KIRVVAEAPTEKRRDE---LFEMA  435
                       : . .* :: : :.. ::.* ***     **:  ::  :..          :
                                         Motif IV

9DU5            IKLLEEALKG----------------    456
1WQA            IELLEKALS-----------------    455
1LXT            APLISIALKVSQLQERTGRTAPTVIT    561
9IX8            YSTVSRIVKEAEKK------------    449
                   :.  :.
```

Figure S8. Multiple sequence alignment (MSA) of PGM*Tk* (PDB ID: 9DU5) with PGM*Ph* (PDB ID: 1WQA), PGM*Oc* (PDB ID: 1LXT), and PPM*Tk* (PDB ID: 9IX8) done using Clustal Omega (https://www.ebi.ac.uk/jdispatcher/msa/clustalo; accessed on 30 December 2024). Sequence alignment shows the presence of four signature motifs for PGMs. Motif I and II are conserved in all four aligned sequences while motif III and IV are conserved in PGM*Tk* and PGM*Ph* only. Symbol '*' (asterisk) shows identical residues; a '.' (period) specifies residues with weekly similar properties; a ':' (colon) symbolizes residues with strongly alike properties; and a '-' (hyphen) shows the gap.

Table S1. Bonds in the interfaces between the principal dimer, AD, of PGM_{Tk}.

H- bonds			Salt bridges		
**Chain A**	**Distance (Å)**	**Chain D**	**Chain A**	**Distance (Å)**	**Chain D**
Ala^{140}	2.9	Asn^{15}	Glu^{21}	3.1	Arg^{139}
Ser^{67}	2.9	Asn^{15}	Glu^{21}	3.4	Arg^{139}
Met^{56}	2.9	Ser^{63}	Arg^{139}	2.9	Glu^{21}
Glu^{21}	3.1	Arg^{139}	Arg^{139}	3.5	Glu^{21}
Glu^{21}	3.1	Arg^{139}	Arg^{139}	3.3	Glu^{21}
Asn^{15}	2.6	Ser^{67}	Arg^{139}	2.8	Glu^{21}
Asn^{15}	3.3	Ala^{140}	Lys^{141}	3.5	Glu^{16}
Ser^{63}	2.7	Met^{56}	-	-	-
Lys^{141}	3.1	Glu^{16}	-	-	-

Table S2. Bonds in the interfaces between the principal dimer, BC, of PGM_{Tk}.

H-bonds			Salt bridges		
**Chain B**	**Distance (Å)**	**Chain C**	**Chain B**	**Distance (Å)**	**Chain C**
Ser^{67}	2.8	Asn^{15}	Glu^{21}	3.0	Arg^{139}
Ala^{140}	2.9	Asn^{15}	Glu^{21}	3.6	Arg^{139}
Met^{56}	3.0	Ser^{63}	Glu^{21}	3.6	Arg^{139}
Glu^{21}	3.0	Arg^{139}	Glu^{21}	2.9	Arg^{139}
Glu^{21}	2.9	Arg^{139}	Glu^{16}	3.5	Lys^{141}
Asn^{15}	2.9	Ala^{140}	Arg^{139}	2.8	Glu^{21}
Asn^{15}	2.8	Ser^{67}	Arg^{139}	3.6	Glu^{21}
Ser^{63}	2.7	Met^{56}	Arg^{139}	3.3	Glu^{21}
Arg^{139}	2.8	Glu^{21}	Arg^{139}	2.8	Glu^{21}

Table S3. Hydrogen bonds and salt bridges between protomers A and C of PGM_{Tk}.

H- bonds			Salt bridges		
**Chain A**	**Distance (Å)**	**Chain C**	**Chain A**	**Distance (Å)**	**Chain C**
Ser^{203}	2.7	Asn^{205}	Asp^{209}	3.4	Arg^{195}
Ser^{203}	2.7	His^{207}	Asp^{209}	3.5	Arg^{195}
Asn^{205}	3.3	Ser^{203}	Arg^{195}	3.6	Asp^{209}
Pro^{208}	2.9	Tyr^{192}	Arg^{195}	3.7	Asp^{209}
Asp^{209}	3.4	Arg^{195}	-	-	-
Asp^{209}	3.5	Arg^{195}	-	-	-
Asn^{205}	2.8	Ser^{203}	-	-	-
Ala^{206}	3.2	Ser^{203}	-	-	-
His^{207}	2.7	Ser^{203}	-	-	-
Ser^{203}	3.3	Asn^{205}	-	-	-
Tyr^{192}	2.6	Pro^{208}	-	-	-

Table S4. Hydrogen bonds and salt bridges between protomers B and D of PGM_{Tk}.

H- bonds			Salt bridges		
**Chain B**	**Distance (Å)**	**Chain D**	**Chain B**	**Distance (Å)**	**Chain D**
Ser^{203}	2.7	Asn^{205}	Asp^{209}	3.4	Arg^{195}
Ser^{203}	2.7	His^{207}	Asp^{209}	3.5	Arg^{195}
Ser^{203}	3.8	Ala^{206}	Arg^{195}	3.7	Asp^{209}
Asn^{205}	3.2	Ser^{203}	Arg^{195}	3.2	Asp^{209}
Pro^{208}	2.9	Tyr^{192}	-	-	-
Asp^{209}	3.2	Arg^{195}	-	-	-
Asn^{205}	2.7	Ser^{203}	-	-	-

His^{207}	2.6	Ser^{203}	-	-	-
Ser^{203}	3.3	Asn^{205}	-	-	-
Tyr^{192}	2.6	Pro^{208}	-	-	-
Arg^{195}	3.1	Asp^{209}	-	-	-
Val^{201}	3.2	Pro^{213}	-	-	-
